# Incidence of healthcare-associated infections in a tertiary hospital in Beijing, China: results from a real-time surveillance system

**DOI:** 10.1186/s13756-019-0582-7

**Published:** 2019-08-27

**Authors:** Yuzheng Zhang, Mingmei Du, Janice Mary Johnston, Ellie Bostwick Andres, Jijiang Suo, Hongwu Yao, Rui Huo, Yunxi Liu, Qiang Fu

**Affiliations:** 1 School of Public Health, The University of Hong Kong, Patrick Manson Building (North Wing), 7 Sassoon Road, Hong Kong, China; 2 Department of Infection Management and Disease Control, Chinese PLA General Hospital, No. 28 Fuxing Road, Haidian District, Beijing, China; 3 XingLin Information Technology Company, No. 57 Jianger Road, Binjiang District, Hangzhou, China; 4 China National Health Development Research Center, No.9 Chegongzhuang Street, Xicheng District, Beijing, China; 5 National Center for Healthcare Associated Infection Prevention and Control, Beijing, China

**Keywords:** Healthcare-associated infection, Incidence, Surveillance

## Abstract

**Background:**

To quantify the five year incidence trend of all healthcare-associated infections (HAI) using a real-time HAI electronic surveillance system in a tertiary hospital in Beijing, China.

**Methods:**

The real-time surveillance system scans the hospital’s electronic databases related to HAI (e.g. microbiological reports and antibiotics administration) to identify HAI cases. We conducted retrospective secondary analyses of the data exported from the surveillance system for inpatients with all types of HAIs from January 1st 2013 to December 31st 2017. Incidence of HAI is defined as the number of HAIs per 1000 patient-days. We modeled the incidence data using negative binomial regression.

**Results:**

In total, 23361 HAI cases were identified from 633990 patients, spanning 6242375 patient-days during the 5-year period. Overall, the adjusted five-year HAI incidence rate had a marginal reduction from 2013 (4.10 per 1000 patient days) to 2017 (3.62 per 1000 patient days). The incidence of respiratory tract infection decreased significantly. However, the incidence rate of bloodstream infections and surgical site infection increased significantly. Respiratory tract infection (43.80%) accounted for the most substantial proportion of HAIs, followed by bloodstream infections (15.74%), and urinary tract infection (12.69%). A summer peak in HAIs was detected among adult and elderly patients.

**Conclusions:**

This study shows how continuous electronic incidence surveillance based on existing hospital electronic databases can provide a practical means of measuring hospital-wide HAI incidence. The estimated incidence trends demonstrate the necessity for improved infection control measures related to bloodstream infections, ventilator-associated pneumonia, non-intensive care patients, and non-device-associated HAIs, especially during summer months.

**Electronic supplementary material:**

The online version of this article (10.1186/s13756-019-0582-7) contains supplementary material, which is available to authorized users.

## Background

Healthcare-associated infections (HAI) are regarded as the most common adverse events in health care service delivery. Evidence indicates HAIs lead to prolonged hospital stay, long-term disability, increased antimicrobial resistance, additional financial burden, and even avoidable deaths [1]. The Study on the Efficiency of Nosocomial Infection Control (SENIC) project demonstrated the importance of surveillance to reduce HAI rates, with data indicating 32% of HAIs could be prevented if all hospitals conducted effective infection surveillance and control programs [2].

The majority of international and national studies estimate the burden of HAIs through two methods: point-prevalence surveys and self-report for targeted infections. Studies from the US, Europe and Singapore showed that HAI point-prevalence ranged from 3.2–11.9% [3–6]. The US National Healthcare Safety Network (NHSN), German national nosocomial infections surveillance system (KISS), and the International Nosocomial Infection Control Consortium (INICC) reported targeted HAI data, such as: device-associated infections rates [7–9]. Unlike these two surveillance methods, continuous incidence surveillance can provide real-time information [10]. For the purpose of launching hospital-wide and long-term incidence surveillance with fewer personnel, some different electronic surveillance programs have been developed [10, 11]. The published studies primarily describe the surveillance system components and performance within high-income countries rather than reported HAI incidence [10, 11]. Only a Finland three-year (2011–2013) electronic HAI surveillance study based mainly on antibiotic treatment, indicated HAI incidence, which was 15.8 per 1000 patient-days and 4.9% of all discharged patients [12].

In 2010, a tertiary hospital in Beijing, China developed a real-time nosocomial infection surveillance system (RT-NISS) [13], which collected process data not only from antibiotics administration, but also from multiple hospital information systems, such as microbiological reports, antibiotics administration, diagnosis, and clinical symptoms.

Our primary objective in this study was to describe the incidence trend of all types of HAI and the proportion of specific HAIs over a 5-year study period for the Beijing hospital.

Except for longitudinal trends in HAIs, periodic pattern and seasonal variation of HAIs were observed in some studies [14, 15]. Meanwhile, a regional difference also impacted the seasonal variations, for the surgical wound infections, winter peaks were detected in the USA and summer peaks were reported in Finland [16]. One study in south of China (Guangzhou) reported no obvious seasonal peaks in nosocomial infection [17], however, there exist remarkable variations of the outdoor temperature between south and north in China, no reports of northern China have been published. Thus, our secondary objective was to explore the seasonal variation of HAI incidence.

## Methods

### Setting

The study was conducted in a 3800-bed teaching hospital in Beijing with 135 wards serving approximately 13000 inpatients per month. The hospital provides quaternary care, such as organ transplants, cardiac surgery, and neurosurgery. The hospital conducted real-time HAI surveillance by monitoring all inpatients during their hospital stay. To protect the privacy of the patients, the study excluded sensitive patient identifiers (e.g. name and identification numbers). Ethical approval was obtained from the Medical Ethical Committee of the Chinese PLA General Hospital (approval number 11KMM51).

### Inclusion/exclusion criteria

Inpatients of any age discharged between 1 January 2013 and 31 December 2017 were eligible for the study. Patients in outpatient settings, physical examination centers, and day surgery centers were excluded.

### Data collection

**Fig. 1:**
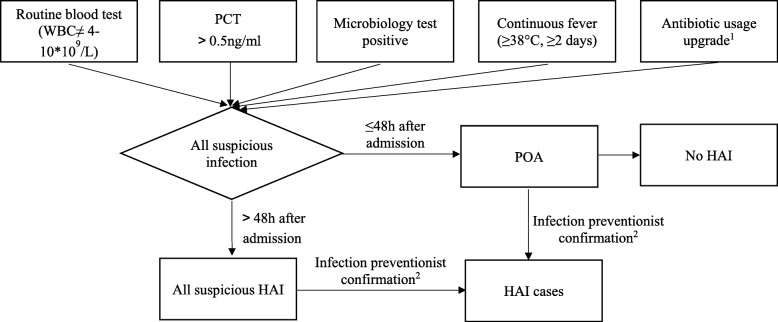
The processing workflow of screening suspicious HAI. Note: WBC, white blood cell; PCT, procalcitonin; HAI, healthcare associated infection; POA, present on admission, the date of event of the site-specific infection criterion occurs on the calendar day 1 and 2 after admission. 1. Antibiotic usage upgrade: upgrade means antibiotics change from the lower to higher level, antibiotics were classified into three levels: un-restricted, restriction, special level. 2. The computer algorithm set > 48 h as one of HAI inclusion criteria, however, during the process of infection preventionist confirmation, few infection cases occurred within 48 h admission were also classified into HAIs, such as: neonate acquired infections from delivery, infections present on admission if related to a prior hospitalization, the device operation at day 1 or day 2

The electronic surveillance system relies on routine process data collected in different hospital databases. RT-NISS has three stages of data collection: data extraction, semi-automated screening, suspicious cases confirmation. (1) The RT-NISS extracts all inpatient infection-related data from four hospital databases: the hospital information system (HIS) (e.g. demographics, antibiotics, surgery data), the laboratory information system (LIS) (e.g. microbiology, routine test results), the radiology information system (RIS) (e.g. radiology results), and the electronic medical record (EMR) (e.g. diagnosis). (2) Suspicious infections are screened by multiple indexes based on the microbiological report, antibiotics administration record, serological and molecular test reports, and body temperature record. Different types of HAI have specific screening strategies (Additional file 1: Figures S1–S4). (3) RT-NISS daily automatic alerts the infection preventionists about 40–50 new suspicious HAIs (occurring at least 48 h after admission) and present on admission (POA, infections present within 48 h). The infection preventionists manually review medical records and confirm suspicious cases according to the criteria. Complex cases are confirmed after discussion with clinicians [18] (Fig. 1). This study exported data for all infected cases confirmed by the infection preventionists and all inpatient information from the RT-NISS.

### Case definition

HAIs are infections acquired more than 48 h after admission, and not present or incubated at admission. In this study, HAIs also include the following situations: first, neonate acquired infections from delivery. Second, infections present on admission if related to a prior hospitalization, namely patient discharge and readmit to the same hospital, and the time interval between discharge and readmission less than 1 days. Third, if an invasive device (central venous catheter, urinary catheter, ventilator) was placed on day 1 or day 2 resulting in any element of infection criteria present with 48 h, and all elements of infection criteria first present together on/after day 3 [19, 20].The *Nosocomial Infection Diagnostic Criteria (2001)* published by the National Health Commission of the People’s Republic of China (NHC) provided the HAI case definition for the study. The NHC criteria was based on the United States (US) *CDC Definitions for Nosocomial Infections (1988)* [21], however, the China and US criteria differ for certain types of infection as indicated in Additional file 1: Table S1 [22, 23]. When inpatients had more than one infection episode, an episode occurring at a different body site (excluding migratory sepsis lesions) or a different type of organism was considered as a new episode (excluding contamination and mixed infection).

### Data analysis

Relative proportions were calculated as each infection site versus all HAIs per year. Incidences of devices-associated infections (central line-associated bloodstream infection, ventilator-associated pneumonia, and catheter-associated urinary tract infection) were calculated as infections per 1000 device days, surgical site infections were calculated as the number of infections per 1000 procedures. Other incidence rate of HAI was defined as the number of new HAIs per 1000 patient-days (Eq. 1). Numerator data of devices-associated infections were available in 2015–2017.

We adjusted incidence rates for age and sex by direct standardization, using the data in 2017 as the standard population. Given the large sample size, most statistical tests of *p*-values indicated statistically significant differences, so we used effect size instead of *p*-values for demographic information. Cramér’s V and Cohen’s d were used to test the nominal and continuous variables’ effect size, respectively. We modeled the incidence of HAIs using negative-binomial regression (incorporating over dispersion), with log number of patient days as offset, and estimated the relative increase in incidence per year in 2017 as the incidence rate ratios (IRR). The IRR of seasonal variation were calculated for the summer (June 1 – August 31) and nine remaining months (September 1–May 30), separately. A 95% confidence intervals (95% CI) for each incidence rate and incidence rate ratio about seasonal variation were estimated based on Poisson distribution. *P* < 0.05 was considered statistically significant. Data analysis was performed using R (version 3.3.2).

1 $$ HAI\kern0.5em incidence\kern0.5em \left( per\kern0.5em 1000\kern0.5em patient\kern0.5em -\kern0.5em days\right)\kern0.5em =\kern0.5em \frac{\mathrm{Number}\kern0.5em \mathrm{of}\kern0.5em \mathrm{HAIs}}{1000\kern0.5em \mathrm{patients}\kern0.5em \mathrm{days}} $$

## Results

### Patient population

633990 unique inpatient admissions were eligible for inclusion (Table 1). In HAI group, male (61.63%) accounted for larger proportions, and the HAI patients’ age was older. However, the ratio of women acquired urinary tract infection were higher, the young age group (≤17) had a relative higher incidence of respiratory tract infections and bloodstream infections (Additional file 1: Figure S5). Owing to the large sample size, the *p*-values of all demographic factors were less than 0.05, however, length of stay had a large effect size, which was more than 0.8. Compared to the group without HAI, the HAI group’s length of stay was much longer (25.05 vs. 7.10). The proportion of Non-intensive care unit (ICU) patients with HAI (87.46%) was much higher than ICU patients (12.54%). Among patients with HAI, 31.39 and 92.92% received surgery and antibiotics, respectively. For the device-associated operation in HAI group, 58.96, 53.61, and 25.95% of patients received a central venous catheter, urinary catheter, and ventilator, respectively.

**Table 1:** Demographic and clinical characteristic of all discharged patients: 2013–2017

Characteristic	Without HAI (*N* = 615132)	With HAI (*N* = 18768)	Effect size^a^
Sex (n, %)			0.03
Male	328942 (53.48)	11567 (61.63)	
Female	286190 (46.52)	7201 (38.37)	
Age (mean, SD)	49.72 (18.85)	53.39 (22.99)	0.17
Length of stay (median, IQR)	7.10 (7.74)	25.05 (20.74)	0.83
Wards (n, %)			0.09
ICU	19055 (3.10)	2353 (12.54)	
Non-ICU	596077 (96.90)	16415 (87.46)	
Receiving antibiotics (n, %)	248348 (40.37)	17440 (92.92)	0.18
Receiving surgery (n, %)	198558 (32.28)	5892 (31.39)	0.00
Device-associated operation (n, %)			
Central venous catheter	157778 (25.65)	11065 (58.96)	0.13
Urinary catheter	170721 (27.75)	10061 (53.61)	0.10
Ventilator	22390 (3.64)	4871 (25.95)	0.19

*SD* standard deviation, *IQR* interquartile range, *ICU* intensive care unit. ^a^Effect size cutoffs: small (0.2), medium (0.5) and large (0.8), Cramér’s V and Cohen’s d were used to test the nominal and continuous variables’ effect size, respectively

### Incidence and distribution of HAI

During the 5-year surveillance period, 23361 HAI cases were identified, including 15470 patients (82.43%) with one episode and 3298 patients (17.57%) with more than one episode of HAI (Table 2). The most common types of HAI were respiratory tract infections (10123, 43.80%), followed by bloodstream infections (3678, 15.74%), urinary tract infections (2965, 12.69%), surgical site infections (2005, 8.58%), and gastrointestinal infections (1735, 7.43%). From 2015 to 2017, device-associated operations accounted for around 11.95–13.79% of all HAIs, including 519 episodes ventilator-associated pneumonia (8.17% of respiratory tract infections), 284 central line-associated bloodstream infections (11.58% of bloodstream infections), and 1092 catheter-associated urinary tract infections (56.90% of urinary tract infections).

**Table 2:** Relative proportion by infection site (number of HAI cases, %), 2013–2017

HAI types	2013	2014	2015	2016	2017	Total
RTI	2014 (45.24)	1866 (44.89)	2158 (42.89)	2178 (43.15)	2015 (43.12)	10231 (43.80)
VAP	–	–	151 (3.00)	186 (3.68)	182 (3.89)	–
BSI	572 (12.85)	653 (15.71)	784 (15.58)	826 (16.36)	843 (18.04)	3678 (15.74)
CLABSI	–	–	94 (1.87)	106 (2.10)	84 (1.80)	–
UTI	544 (12.22)	502 (12.08)	653 (12.98)	710 (14.06)	556 (11.9)	2965 (12.69)
CAUTI	–	–	356 (7.08)	404 (8.00)	332 (7.10)	–
SSI	194 (4.36)	242 (5.82)	551 (10.95)	497 (9.85)	521 (11.15)	2005 (8.58)
GI	340 (7.64)	289 (6.95)	367 (7.29)	391 (7.75)	348 (7.45)	1735 (7.43)
SST	152 (3.41)	137 (3.3)	202 (4.02)	130 (2.58)	119 (2.55)	740 (3.17)
CNS	125 (2.81)	158 (3.8)	11 (0.22)	18 (0.36)	13 (0.28)	325 (1.39)
OCI	61 (1.37)	36 (0.87)	53 (1.05)	39 (0.77)	27 (0.58)	216 (0.92)
REPR	3 (0.07)	4 (0.1)	7 (0.14)	1 (0.02)	2 (0.04)	17 (0.07)
BJ	8 (0.18)	0	1 (0.02)	0	2 (0.04)	11 (0.05)
CVS	0	0	1 (0.02)	1 (0.02)	0	2 (0.01)
Others	439 (9.86)	270 (6.5)	243 (4.83)	257 (5.09)	227 (4.86)	1436 (6.15)
Total	4452 (100)	4157 (100)	5031 (100)	5048 (100)	4673 (100)	23361 (100)

Note: within one admission, 15470 patients with one episode of HAI, 3298 patients with more than one episode of HAI. *RTI* respiratory tract infection, *VAP* ventilator-associated pneumonia, BSI bloodstream infection, *CLABSI* central line-associated bloodstream infection, *UTI* urinary tract infection, *CAUTI* catheter-associated urinary tract infection, *SSI* surgical site infection, *GI* gastrointestinal infection, *SST* skin and soft-tissue infection, *CNS* central nervous system infection, *OCI* oral cavity infection, *REPR* Reproductive tract infection, *BJ* bone and joint infection, *CVS* cardiovascular system infection. The data (−) were not available

**Fig. 2:**
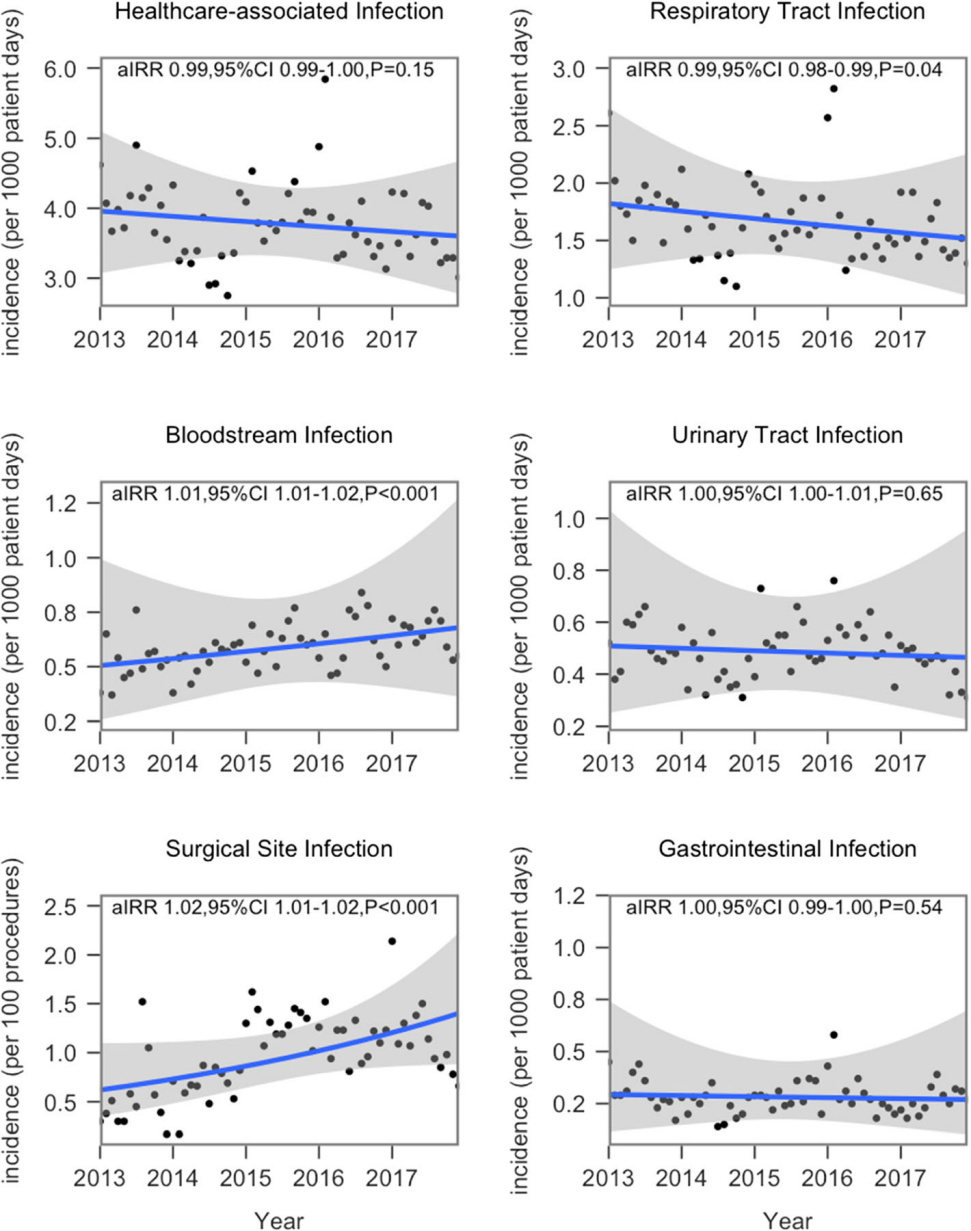
Monthly HAI incidence, 2013–2017. Note: aIRR, annual incidence rate ratio. Incidence rate was standardized by age and sex of 2017, the change of time trends was tested by negative binomial regression. Surgical site infections were calculated as the number of infections per 1000 procedures. Other incidence rate of HAI was defined as the number of new HAIs per 1000 patient-days

The 5-year incidences of HAIs by specific infection site were provided in Fig. 2 and Table 3. The adjusted HAIs decreased slightly from 2013 (4.10 per 1000 patient days) to 2017 (3.62 per 1000 patient days), and negative binomial regression indicated the trends had no statistical significance (IRR 0.99, 95% CI 0.99–1.00). It also revealed a declining trend in respiratory tract infections, an increasing trend in bloodstream infections (IRR 1.01, 95% CI 1.01–1.02) and surgical site infections (IRR 1.02, 95% CI 1.01–1.02), those changes were statistically significant (*P* < 0.05). The incidence rate of urinary tract infections, gastrointestinal infections, and device-associated infections remained unchanged.

**Table 3:** Incidence of HAI in different departments (per 1000 patient-days), 2013–2017

Infection type	Incidence (95% CI)					aIRR (95% CI) ^b^	*P*-value^a^
	2013	2014	2015	2016	2017		
All HAI	4.10 (3.97, 4.22)	3.41 (3.31, 3.52)	3.96 (3.85, 4.07)	3.78 (3.68, 3.89)	3.62 (3.52, 3.73)	0.99 (0.99–1.00)	0.15
RTI	1.86 (1.78, 1.94)	1.52 (1.46, 1.60)	1.70 (1.63, 1.77)	1.63 (1.56,1.70)	1.56 (1.49, 1.63)	0.98 (0.98–0.99)	0.04*
VAP	–	–	6.44 (5.47, 7.57)	7.09 (6.13, 8.21)	7.40 (6.38, 8.57)	1.00 (1.00–1.02)	0.11
Non-VAP	–	–	1.56 (1.50, 1.63)	1.50 (1.43, 1.56)	1.41 (1.35, 1.48)	0.99 (0.98–0.99)	0.02*
BSI	0.53 (0.49, 0.58)	0.54 (0.50, 0.58)	0.62 (0.58, 0.66)	0.62 (0.58, 0.66)	0.65 (0.61, 0.70)	1.01 (1.01–1.02)	< 0.001*
CLABSI	–	–	0.33 (0.27, 0.41)	0.37 (0.30, 0.44)	0.31 (0.25, 0.39)	1.00 (0.98–1.00)	0.81
Non-CLABSI	–	–	0.53 (0.50, 0.57)	0.54 (0.50, 0.58)	0.58 (0.55, 0.63)	1.00 (0.99–1.01)	0.15
UTI	0.53 (0.49, 0.58)	0.42 (0.39, 0.46)	0.53 (0.49, 0.57)	0.53 (0.50, 0.58)	0.43 (0.40, 0.47)	1.00 (1.00–1.01)	0.65
CAUTI	–	–	2.65 (2.38, 2.94)	2.94 (2.66, 3.24)	2.53 (2.27, 2.82)	0.99 (0.99–1.00)	0.43
Non-CAUTI	–	–	0.23 (0.21, 0.26)	0.23 (0.20, 0.26)	0.17 (0.15, 0.20)	0.98 (0.97–0.99)	< 0.001*
SSI	0.53 (0.46, 0.61)	0.66 (0.58, 0.74)	1.23 (1.18, 1.40)	1.12 (1.02, 1.22)	1.13 (1.03,1.23)	1.02 (1.01–1.02)	< 0.001*
GI	0.31 (0.28, 0.35)	0.24 (0.21, 0.27)	0.29 (0.26, 0.31)	0.29 (0.26, 0.32)	0.27 (0.24, 0.30)	1.00 (0.99–1.00)	0.54

Note. Adjusted by sex and age with data of 2017. ^a^The change of time trends was tested by negative binomial regression. ^b^*aIRR* = annual incidence rate ratio in 2017. *RTI* respiratory tract infection, *VAP* ventilator-associated pneumonia, *BSI* bloodstream infection, *CLABSI* central line-associated bloodstream infection, *UTI* urinary tract infection, *CAUTI* catheter-associated urinary tract infection, *SSI* surgical site infection, *GI* gastrointestinal infection. *CI* confidence interval. The data (−) were not available

### Seasonal variation

Comparison of incidence rates during the summer months versus the rest of the year revealed a significant overall increase in HAI during the summer (IRR 1.03, 95% CI 1.00 to 1.06) (Table 4). However, the respiratory tract infection, child (age ≤ 17), the female had a lower incidence during summer. Especially, the child subgroup had significant declines in HAI incidence during the summer months compared the rest of the year (IRR 0.87, 95% CI 0.79 to 0.95).

**Table 4:** Incidence rates of HAI during summer (June–August) compared with the rest of the year, 2013–2017

Comparison	IR (95% CI)		IRR (95% CI)
	June–August (2013–2017)	Rest of the year (2013–2017)	IR (summer)/IR (the rest)
Overall	3.82 (3.73, 3.92)	3.71 (3.67, 3.77)	1.03 (1.00, 1.06)
Respiratory tract infection	1.59 (1.53, 1.66)	1.65 (1.62, 1.69)	0.96 (0.92, 1.01)
Bloodstream infection	0.65 (0.61, 0.69)	0.57 (0.55, 0.59)	1.15 (1.07, 1.23)
Urinary tract infection	0.51 (0.48, 0.55)	0.46 (0.44, 0.48)	1.11 (1.02, 1.20)
Surgery site infection	0.33 (0.30, 0.36)	0.32 (0.30, 0.34)	1.03 (0.93, 1.14)
Gastrointestinal infection	0.30 (0.27, 0.33)	0.27 (0.26, 0.29)	1.11 (1 .00, 1.23)
Stratified analyses by sex			
Male	4.27 (4.13, 4.40)	4.05 (3.98,4.13)	1.05 (1.01, 1.09)
Female	3.24 (3.11, 3.38)	3.26 (3.18, 3.34)	0.99 (0.95, 1.04)
Stratified analyses by age			
≤ 17	4.36 (4.01, 4.75)	5.04 (4.79, 5.29)	0.87 (0.79, 0.95)
18–44	3.02 (2.85, 3.19)	2.92 (2.82, 3.02)	1.03 (0.97, 1.10)
45–64	3.08 (2.95, 3.21)	2.93 (2.86, 3.01)	1.05 (1.00, 1.10)
≥ 65	5.77 (5.54, 6.01)	5.50 (5.36, 5.63)	1.05 (1.00, 1.10)

*IR* incidence rate. *IRR* incidence rate ratio. *CI* confidence interval

## Discussion

This study described findings from secondary data analysis of a hospital-wide continuous electronic HAI incidence surveillance system in China. Our results of incidence are similar to a meta-analysis of the HAI point-prevalence in Mainland China, indicating a weighted prevalence of 3.12% (95% CI, 2.94–3.29%) [24]. However, compared to international studies, the incidence rate in our study is relatively low [25, 26]. US researchers estimated the HAI incidence density was 2.6–13 episodes per 1,000 patient-days [25]. One US hospital-wide surveillance reported the incidence rate was 4.59 per 1000 patient days in 2012 [26]. The differences with international rates could be explained by the large denominator (long length of stay) in our study. Due to expansions of insurance coverage to hospital services in 2003 and comprehensive care in 2008 in China, patients preferred to receive in-hospital treatment [27]. Typically, data in 2015 showed that average length of stay in China (9.6 days) was longer than OECD countries (8 days) [28, 29].

Our study documented a much higher proportion of bloodstream infections (15.74%) than the average for China (2.65%) [24], which might be a result of the hospital’s new policy encouraging blood cultures to enhance the detection rate of microbiology test. Even though the rate of respiratory tract infection decreased statistically, the incidence of ventilator-associated pneumonia (7.40 per 1000 patient days in 2017) was obviously higher than US study (1.80 per 1000 patient days in 2012) [26].

For the proportion of device-associated infections, it accounted around only 11–13% of all HAIs in our study, which was lower than published US study (25.38%) [4]. Another US study reported that device-associated infections accounted for 38.7% of pneumonia cases, 62.3% of bloodstream infections, and 77.7% of urinary tract infections [30]. However, ventilator-associated pneumonia and central line-associated bloodstream infections in our study accounted for a much lower proportion of respiratory tract infections (8.17%) and bloodstream infections (11.58%), respectively. The study also showed a low proportion of ICU infections (12.54%), which was apparently lower than the US hospital (33% in 2012) [26].

The current surveillance mainly focused on targeted infections, such as: ICUs, device-associated infections, and surgical site infections. The previous study demonstrated that targeted surveillance missed approximately 50% HAIs [31], our results showed the proportion was even higher. Therefore, our study highlighted the importance of infections control for non-device associated infections and non-ICU inpatient units.

Meanwhile, we observed an overall summer peak in incidence of HAI among adult and elderly. Prior studies showed evidence of seasonal variation with catheter-related bloodstream infections peaking during summer months [32, 33]. Some reports suggested that surgical site infection could be due to a “July effect” explained by staff turnover at teaching institutions [34]. The divergent finding among children may be attributed to the winter peak in respiratory tract infections due to cold weather [35].

A review of HAI electronic surveillance system in developed countries demonstrated that the best performing algorithm included the use of three data sources: positive microbiological results, initiation of antibiotic therapy and diagnosis codes [10]. The RT-NISS synthesized data beyond these three databases to identify suspicious HAI cases. Compared to the golden standard-manual review, validation studies of RT-NISS showed good performance, validating HAIs occurred in July 2011 reported relatively high sensitivity (98.8%) and specificity (93.0%), another validation study reported 71.9% surgical site infections among 3,048 patients could be detected by RT-NISS [18, 36]. Hospital-wide surveillance was ever reported in a US hospital through manual review. However, they invested large human resources, and their annual inpatient admissions was much lower than our hospital [26]. Our electronic surveillance system could provide a reference for healthcare facilities with insufficient human resources.

This study also has some limitations. First, the accuracy of all the data was not validated by manual review of the medical records. However,143,750 suspicious cases (22.67%) were reviewed manually and 0.04% (23361) HAI cases were confirmed by the infection preventionists. Second, The RT-NISS lacks surveillance for surgical site infections after hospital discharge. A systematic review showed that 60% of surgical site infections appeared after discharge^23^. The previous comparison between manual review and the RT-NISS showed that 94% were detected by RT-NISS from current admission and readmission and 18% were detected post-discharge^16^. Third, owing to the updated screening algorithm of the surveillance system, some data were not available or underestimated at the beginning years. Such as: the data of device-associated infections were not available, and the increasing rate of surgical site infection in 2015 was mainly caused by the system updated, some infections without fever and microbiology test were identified through the antibiotic usage upgrade. Forth, the incidence rates in current study were estimated without any risk adjustment strategies. The importance of risk-adjusted measurement was mentioned in different studies, such as: NHSN risk-adjusted measures for surgical site infection [37], McCabe and Jackson score for survival of patients with gram-negative bacteraemia [38]. We will add the procedure-specific risk adjustment variables and disease severity assessment to the surveillance system in the future. Fifth, lack of data about infection control interventions during the study period, relationships between the incidence rate and interventions should be further explored. As the surveillance system only collected the frequency of antimicrobial usage and their resistance pattern, a lack of detailed drug utilization data in this study, such as: defined daily dose (DDD). More comprehensive information about antimicrobial usage need to be collected in the future surveillance.

## Conclusions

This study, even a single center study, shows how a hospital-wide continuous electronic surveillance system based on existing hospital electronic databases could provide a practical means for measuring hospital-wide HAI incidence. Our study showed the incidence slightly decreased over the last five years, and it provided an evidence of the necessity for infection control in bloodstream infections, ventilator associated pneumonia, non-ICU and non-device-associated infections. The summer peak also showed the interventions on specific HAIs should be emphasized.

## Additional files

Additional file 1: **Table S1.** Differences of HAI criteria between China and US. **Figure S1.** Flow chart of screening surgical site infections. **Figure S2.** Flow chart of screening bloodstream infections. **Figure S3.** Flow chart of screening urinary tract infections. **Figure S4.** Flow chart of screening respiratory tract infections. **Figure S5.** HAI incidence rate by age group. (DOCX 569 kb)

## Data Availability

The datasets generated during the current study are not publicly available, to avoid disclosure of the individual privacy of the patients. However, they are available from the corresponding author (LIU Yunxi: liuyunxi301@qq.com) on reasonable request.

## Abbreviations

BJ: Bone and joint infection
BSI: Bloodstream infection
CAUTI: Catheter-associated urinary tract infection
CI: Confidence interval
CLABSI: Central line-associated bloodstream infection
CNS: Central nervous system infection
CVS: Cardiovascular system infection
GI: Gastrointestinal infection
HAI: Healthcare-associated infection
ICU: Intensive care unit
INICC: International Nosocomial Infection Control Consortium
IQR: Interquartile range
IR: Incidence rate
IRR: Incidence rate ratio
KISS: Krankenhaus Infektions Surveillance System (German national nosocomial infections surveillance system)
NHSN: National Healthcare Safety Network
OCI: Oral cavity infection
PCT: Procalcitonin
REPR: Reproductive tract infection
RTI: Respiratory tract infection
SD: Standard deviation
SSI: Surgical site infection
SST: Skin and soft-tissue infection
UTI: Urinary tract infection
VAP: Ventilator-associated pneumonia
WBC: White blood cell
