# Supplementary material for: Incidence of healthcare-associated infections in a tertiary hospital in Beijing, China: results from a real-time surveillance system
Source: Antimicrob Resist Infect Control. 2019 Aug 27;8:145. doi: 10.1186/s13756-019-0582-7 (PMC6712817; doi:10.1186/s13756-019-0582-7)
Supplement: Supplementary file 1 — Table S1. Differences of HAI criteria between China and US. Figure S1. Flow chart of screening surgical site infections. Figure S2. Flow chart of screening bloodstream infections. Figure S3. Flow chart of screening urinary tract infections. Figure S4. Flow chart of screening respiratory tract infections. Figure S5. HAI incidence rate by age group. (DOCX 569 kb) [file 13756_2019_582_MOESM1_ESM.docx]

**Additional file 1**

Additional supporting information may be found in the online version of this article:

**Table S1** Differences of HAI criteria between China and US

**Figure S1** Flow chart of screening surgical site infections

**Figure S2** Flow chart of screening bloodstream infections

**Figure S3** Flow chart of screening urinary tract infections

**Figure S4** Flow chart of screening respiratory tract infections

**Figure S5** Figure S5 HAI incidence rate by age group

**Table S1 Differences of HAI criteria between China and US**

| **China** | **US** |
| --- | --- |
| Respiratory system infection:   - Upper respiratory tract infection (URTI) - Lower respiratory tract infection (LRTI, including pneumonia) - Pleural space infection | - Pneumonia infection - Ventilator-associated events - Lower Respiratory system infection, other than pneumonia |
| Urinary tract infection | - Urinary tract infection - Urinary system infection (kidney, ureter, bladder, urethra, or tissue surrounding the retroperitoneal or perinephric space) |
| Bloodstream infection: include secondary infection | Bloodstream infection: exclude secondary infection |
| Oral cavity infection | Eye, Ear, Nose, Throat, Mouth infection (EENT):   - Conjunctivitis - Eye, other than conjunctivitis - Ear, mastoid - Oral cavity (mouth, tongue, or gums) - Sinusitis - Upper respiratory tract, pharyngitis, laryngitis, epiglottitis |

**Note:** China and US current criteria include 12 and 14 major types HAI respectively, 7 types are consistent, 4 types are differences.

Surgery performed during admission

Wound culture collected with 30 days

Positive culture result

Negative culture result

No wound culture collected with 30 days

Diagnosis name or ICD-9-CM code for post-operation infection

Diagnosis name or ICD-9-CM code for post-operation infection

＜30 days readmission with antibiotics

**Figure S1 Flow chart of screening surgical site infections (SSI)**

Based on the general screen strategy, the specific flow of processing information with multiple data sources for SSI. Gray boxes represent the number of patient discharges identified as suspicious SSI.

Blood/CVC tip culture

Negative culture

Positive culture

Secondary to other infection

(Respiratory, wound, urine)

Primary BSI

CLABSI

Non-CLABSI

**Figure S2 Flow chart of screening bloodstream infections**

Gray boxes represent the number of patient discharges identified as suspicious bloodstream infections.

CVC: Central venous catheter; BSI: bloodstream infections; CLABSI: central line-associated blood stream infection

Urinalysis

(WBC: male≥5/hpf;

female ≥ 10/hpf)

Urinary culture

(≥10^5^ cfu/ml)

No UTI

UTI

≤2 species in urinary culture

CAUTI

Non-CAUTI

**Figure S3 Flow chart of screening urinary tract infections**

Gray boxes represent the number of patient discharges identified as suspicious urinary tract infections.

UTI, urinary tract infections; CAUTI: catheter-associated urinary tract infections

X-ray/CT-scan

(New or progressive changes:

Infiltrate, consolidation, cavitation)

Positive sputum culture

Pleural fluid analysis

(WBC≥10^9^/L)

RTI

Receiving mechanical ventilation more than 48h;

OR after removing ventilator within 48h

VAP

Non-VAP

**Figure S4 Flow chart of screening respiratory tract infections**

Gray boxes represent the number of patient discharges identified as suspicious respiratory tract infections.

RTI, respiratory tract infections; VAP: ventilator associated infection


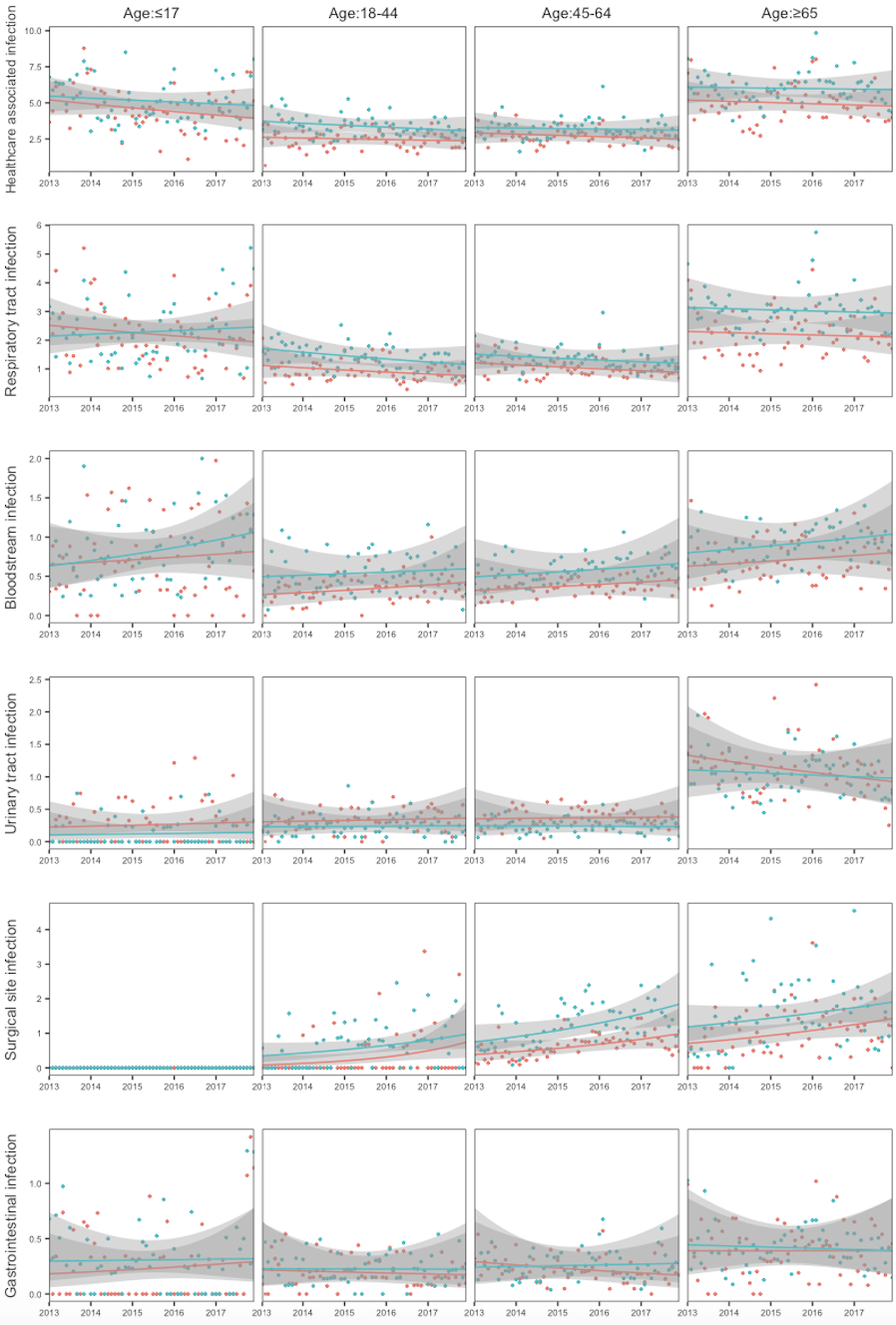


**Figure S5 HAI incidence rate by age group.** Fitted lines are for men (blue) and women (red).
